# Dye-Sensitized Multiple Exciton Generation in Lead Sulfide Quantum Dots

**DOI:** 10.1021/jacs.2c07109

**Published:** 2022-08-18

**Authors:** Zhiyuan Huang, Matthew C. Beard

**Affiliations:** Chemistry & Nanoscience Center, National Renewable Energy Laboratory, Golden, Colorado 80401, United States

## Abstract

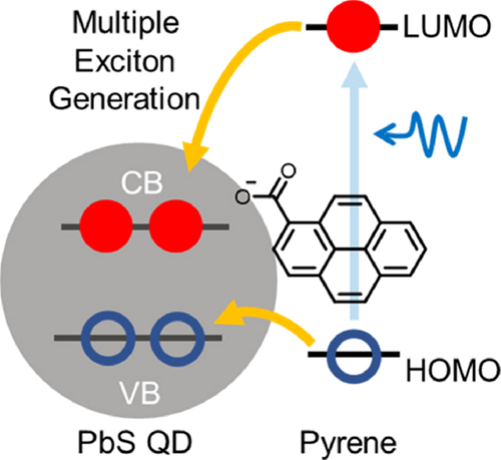

Multiple exciton generation (MEG), the generation of multiple excitons from the absorption of a single high-energy photon, is a strategy to go beyond the limiting efficiencies that define current-day solar cells by harvesting some of the thermalization energy losses that occur when photons with an energy greater than the semiconductor bandgap are absorbed. In this work, we show that organic dyes can sensitize MEG in semiconductor quantum dots (QDs). In particular, we found that surface-anchored pyrene ligands enhanced the photon-to-charge carrier quantum yield of PbS QDs from 113 ± 3% to 183 ± 7% when the photon energy was 3.9 times the band gap. A wavelength dependence study shows that the enhancement is positively correlated with the pyrene absorptivity. Transient absorption and steady-state photoluminescence measurements suggest that the MEG sensitization is based on an initial fast electron transfer from the pyrene ligands to the PbS QDs producing hot-electrons in the QDs that subsequently undergo MEG. This work demonstrates that hybrid and synergistic organic/inorganic interactions can be a successful strategy to enhance MEG.

## Introduction

The power conversion efficiency of state-of-the-art single-junction solar cells has been steadily approaching the limiting efficiency that governs all modern-day solar cells.^1^ The goal of the next generation of photovoltaic absorbers is to enable strategies that can go beyond current limitations, such as bypassing unnecessary thermalization losses, and thereby redefine the limiting efficiency, that is, begin to approach the Ross–Nozik theoretical limiting efficiency.^2^ One of the major energy loss pathways that define current limiting efficiencies is the thermalization of hot-charge carriers that are produced when the semiconductor absorbs photons with energies that are larger than its fundamental bandgap (*E*_g_). Multiple exciton generation (MEG),^3−5^ or carrier multiplication, describes the conversion of one high-energy photon into multiple electron/hole pairs, or excitons, when the energy of the photon is at least 2 × *E*_g_ and is one way to harvest some of the otherwise wasted photon energy. Simulation results show that MEG can boost the theoretical efficiency of single-junction solar cells up to ∼45% at 1-sun and > ∼80% at 500× concentration.^6,7^ In the MEG process, Coulombic collisions between hot-carriers and electrons that remain in their ground state can generate extra electron–hole pairs while de-exciting the hot-carriers. However, MEG competes with hot-carrier cooling, where the hot-carriers lose their excess energy via emission of phonons rather than generating additional excited electrons. In 2002, Nozik suggested that quantum confined semiconductors (e.g., quantum dots (QDs), nanorods, 2D semiconductors, etc.) are promising candidates for MEG because (1) the discrete energy levels that result from quantum-confinement can slow down the carrier cooling process; (2) momentum conservation is relaxed in highly quantum-confined systems where crystal momentum is ill-defined, increasing the final density of states; and (3) the quantum confined nature of the nanostructures enhances the Coulombic collisions that drive the MEG process, thus, enhancing the MEG relaxation pathway over hot-carrier cooling.^8^ Following this work, MEG has been demonstrated in a series of low-dimensional quantum confined nanomaterials, for example, QDs, nanorods, 2D semiconductors, and so on.^4^

While a number of examples demonstrate that MEG can be operational in photovoltaic architectures^9,10^ and photoelectrochemical reactions,^11^ showing quantum efficiencies exceeding 100% in the collected current or photogenerated molecules, systems with a higher MEG energy efficiency, that is, systems that exhibit a low threshold photon energy and high quantum yields (QYs) are required in order to make the largest impact on solar energy conversion. Ideally, the threshold energy of MEG is 2 × *E*_g_, and the QY should reach 200% when the photon energy reaches 2 × *E*_g,_ 300% for 3 × *E*_g_ photons, and so forth. It is worth noting that 2D van der Waals layered semiconductors can undergo MEG with a near-perfect efficiency,^12^ but the relatively weak optical absorption^13−15^ and fast decay of carriers limit their utility as light absorbers in solar energy conversion strategies. On the other hand, the enhanced MEG observed in typical low-dimensional nanostructures such as single-component PbE (E = S, Se, or Te) QDs is less than 120%, even with 3 × *E*_g_ photons, and the threshold photon-energies are greater than 2.5 × *E*_g_. There are two major reasons that limit the MEG efficiencies in these systems: one is the competition of MEG with hot-carrier cooling; the other is that the excess photon energy (*h*ν-*E*_g_) is equally divided between the electrons and holes because of the nearly mirror-like symmetric conduction band (CB) and valence band (VB) found in PbE semiconductors.^5^ These concerns have been addressed by the development of specific heteronanostructures designed to overcome one or both of these limitations. For example, Cirloganu et al.^16^ reported a four-fold enhancement in the enhanced QYs when using PbSe-CdSe core–shell QDs compared with the single-component PbSe QDs and a reduction of threshold photon energy to 2.18 × *E*_g_. With PbS-CdS Janus QDs, Kroupa et al.^17^ reported a staircase-like response (i.e., the MEG onset was close to 2 × *E*_g_ and rose abruptly, but only to ∼120%, and then rose again near 3 × *E*_g_) that indicated ∼25% of the absorbed photons generated carriers that then underwent MEG with an efficiency of 0.98, but the remaining 75% of the absorbed photons produces carriers with a lower MEG efficiency. The CB–VB symmetry in single-component PbE QDs is broken in these heteronanostructures, and the decoupled shell states or interfacial states were shown to slow down the hot-carrier cooling. Both effects increase the MEG energy efficiency. However, the electronic states of these heteronanostructures are typically ill-defined. For example, the shell states in core–shell QDs are dependent on the uniformity of the shell growth, and the interfacial states in Janus QDs are correlated with the ratio of the two components; both are difficult to control precisely in experiments.

**Figure 1:**
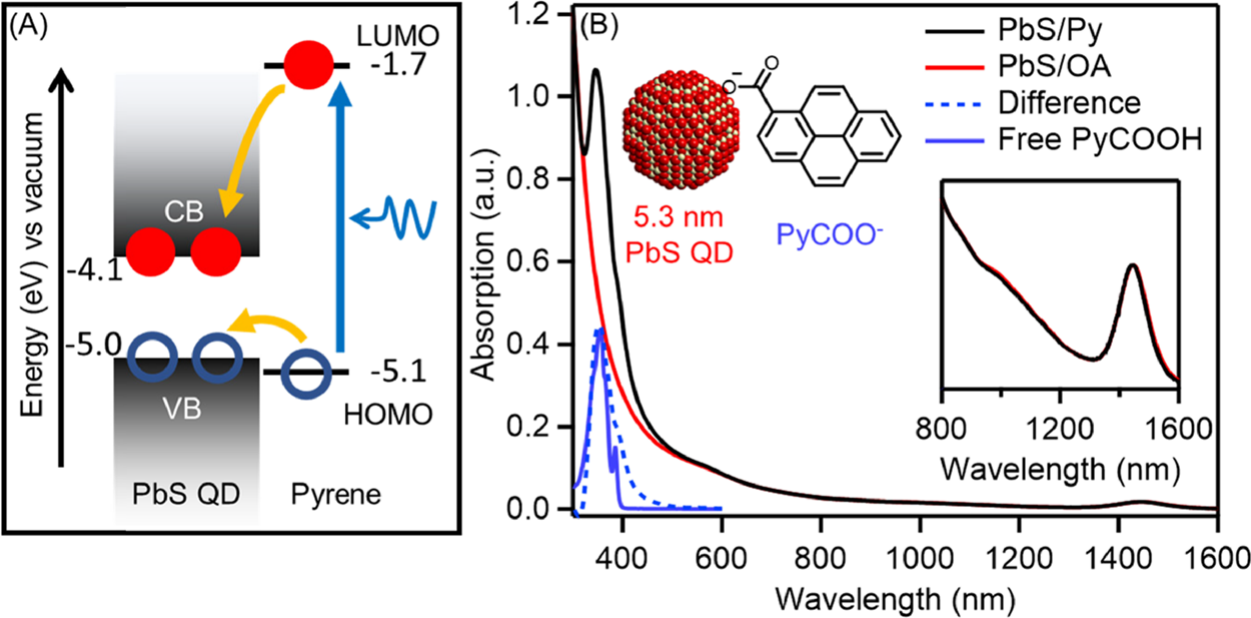
(A) Schematic illustration of electronic states in PbS QDs with surface-anchored pyrene. (B) Absorption spectra of PbS QDs capped with oleate (PbS/OA, black solid) and pyrene carboxylate (PbS/Py, red solid), the difference spectrum (blue dashed) by subtracting PbS/OA from PbS/Py, and the free pyrene carboxylic acid (blue solid). Inset: the first excitonic peak of PbS QDs. All samples are dissolved in tetrachloroethylene.

Organic dyes are good alternatives to sensitize MEG. Compared with inorganic shells, organic dyes possess well-defined electronic states and comparable absorption coefficients and can easily functionalize QDs through ligand exchange reactions. In this work, we show that pyrene as a photosensitizer enhances MEG when attached to PbS QDs. The idea is presented in Figure 1A; 5.3 nm PbS QDs are functionalized with pyrene carboxylate with a highest occupied molecular orbital (HOMO)–lowest unoccupied molecular orbital (LUMO) gap of 3.8 × *E*_g_, where *E*_g_ is the band gap of PbS QDs. MEG is initiated by photoexcitation of the pyrene ligands, followed by a rapid energy transfer into the QDs to produce an asymmetric excitation, that is, a hot-electron that contains all of the excess energy, and a hole near the top of the VB, that is, with ∼ no excess energy. As the energy gap between the LUMO^18^ of pyrene and the CB minimum^19^ is higher than *E*_g_, the hot-electron produced in the QDs can undergo MEG to populate another electron–hole pair. With transient absorption (TA) spectroscopy, we show that surface-anchored pyrene enhances the QY from 113 ± 3% to 183 ± 7% when pumping at 3.9 × *E*_g_.

## Results and Discussion

The PbS-pyrene complex (denoted here as PbS/Py) is prepared through a ligand exchange reaction. First, 5.3 nm-diameter PbS QDs were synthesized following reported methods.^20^ Then, the as-synthesized PbS QDs terminated with oleate (denoted here as PbS/OA) were ligand-exchanged with 1-pyrenecarboxylic acid (see Experimental Section) in solution. Efficient energy transfer processes require close donor-acceptor proximity, that is, free 1-pyrenecarboxylic acid cannot transfer energy to the PbS QDs efficiently and should therefore be completely removed. After ligand exchange, the PbS/Py complex was washed three times, and proton nuclear magnetic resonance (NMR) spectroscopy (Figure S1) confirms that no free ligands remained in the sample. The linear absorption spectrum of PbS/Py is displayed in Figure 1B. The difference spectrum by subtracting the spectrum of PbS/OA from PbS/Py represents the contribution of bound pyrene (Figure 1B, blue-dashed trace). Compared with the free 1-pyrenecarboxylic acid (Figure 1B, blue-solid trace), the bound pyrene ligand shows peak broadening, which is evidence of covalent bonding to the PbS QDs. No shifts are observed for either pyrene or the first excitonic peak of PbS QDs after ligand exchange, indicating that the pyrene-PbS electronic coupling is relatively weak.^21^ The first excitonic peak of PbS/Py well overlaps with that of PbS/OA without peak broadening, which means that the colloidal stability and size distribution are maintained after the ligand exchange. This is demonstrated by transmission electron microscopy (TEM) images of QDs before and after ligand exchange (Figure S2).

**Figure 2:**
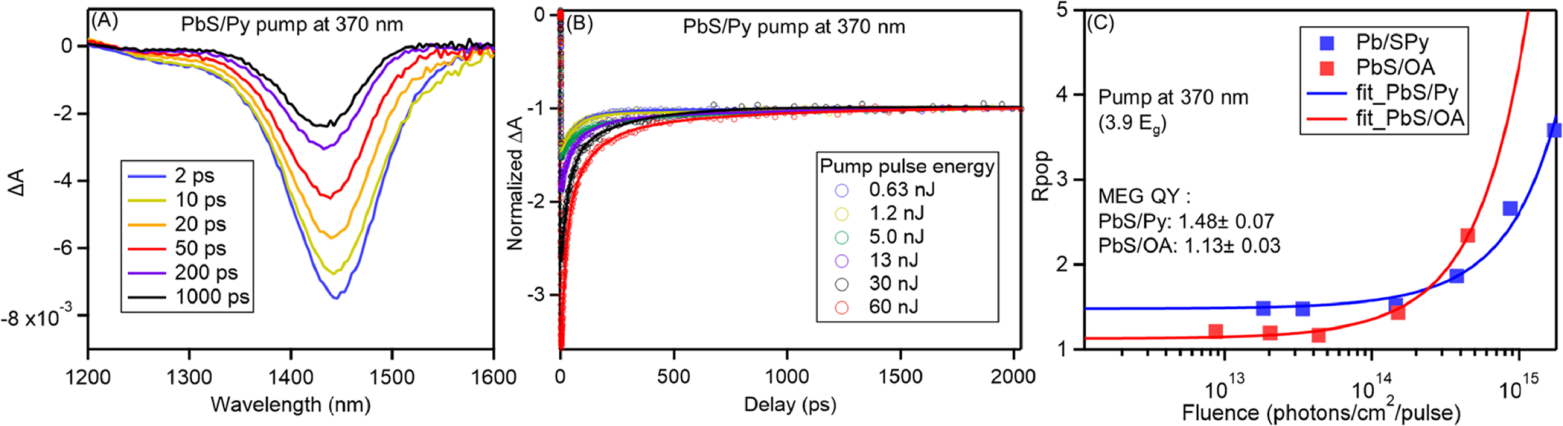
Representation of the TA spectra and MEG analysis. (A) The TA spectra showing the first exciton bleach of PbS/Py at different delay time. The pump is at 370 nm with a pulse energy of 60 nJ. (b) Kinetics at the first exciton bleach minimum of PbS/Py with different pump pulse energy after normalization at tails. The solid curves are the fittings to the data points used to extract Δ*A* at different delay time. (C) *R*_pop_, Δ*A* minimum divided by Δ*A* at 2000 ps in (B), versus the pump photon fluence at 370 nm for PbS/Py (blue) and PbS/OA (red). The solid curves are fittings with eq 1 to extract MEG QYs.

MEG of PbS/Py and PbS/OA was characterized using TA spectroscopy. Here, we employed the first exciton bleach (Figure 2A) to track the exciton population. In PbS QDs, the major decay pathway of biexcitons is through Auger recombination:^22^ one electron–hole pair recombines nonradiatively giving its energy to the other exciton and promoting it to a higher energy state followed by fast relaxation of the Auger-generated hot-exciton. Therefore, the exciton bleach kinetics includes two distinct decay pathways, the fast Auger recombination of biexcitons followed by the slow single exciton decay. The ratio of the exciton bleach before and after the Auger recombination, *R*_pop_, is determined by the exciton multiplicity. Prior to measuring *R*_pop_, we first investigated the biexciton lifetime. With low pump photon-energy, 800 nm (<*E*_g_), but a large fluence each QD can absorb more than one photon to generate multiple excitons, but the photon energy is too low to be able to create carriers that can undergo MEG. In Figure S3, evident fast biexciton decay is observed followed by the long-lived single exciton decay. The mono-exponential fitting to the initial fast decay yields a biexciton lifetime of 79.2 ± 1.2 ps for PbS/OA and 81.8 ± 2.7 ps for PbS/Py, indicating that the binding of pyrene ligands does not significantly change the QD biexciton lifetime. The minimal TA decay over 1 ns for 800 nm pumping at low fluence (Figure S3A,B) and the mono-exponential form of the decay transient, consistent between high fluence 800 nm pumping and 370 nm pumping (Figure S3C), both suggest no significant photocharging effect in the TA measurements. In Figure 2B, we take the bleach at 2 ns to represent the exciton population after Auger recombination (Δ*A*_long_), and the Δ*A* minimum at early decay time to represent the exciton population before Auger recombination (Δ*A*_short_). The ratio of Δ*A*_short_ and Δ*A*_long_ provides the exciton multiplicity, *R*_pop_, and the MEG enhanced QYs can be obtained by measuring *R*_pop_ at different pump fluences and fitting with:^5,23^
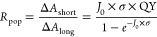
1where *J*_0_ is the photon fluence of the pump pulse, σ is the absorption cross section at the pump wavelength, and at low fluence where (1 – *e*^–*J*_0_σ^) → *J*_0_σ, then *R*_pop_ → QY. The results are plotted in Figure 2C. With the pump at 370 nm, we found a QY for the PbS/Py to be 148 ± 7%, 35% higher than 113 ± 3% found for the PbS/OA sample, demonstrating the sensitization effect of the pyrene ligands.

**Figure 3:**
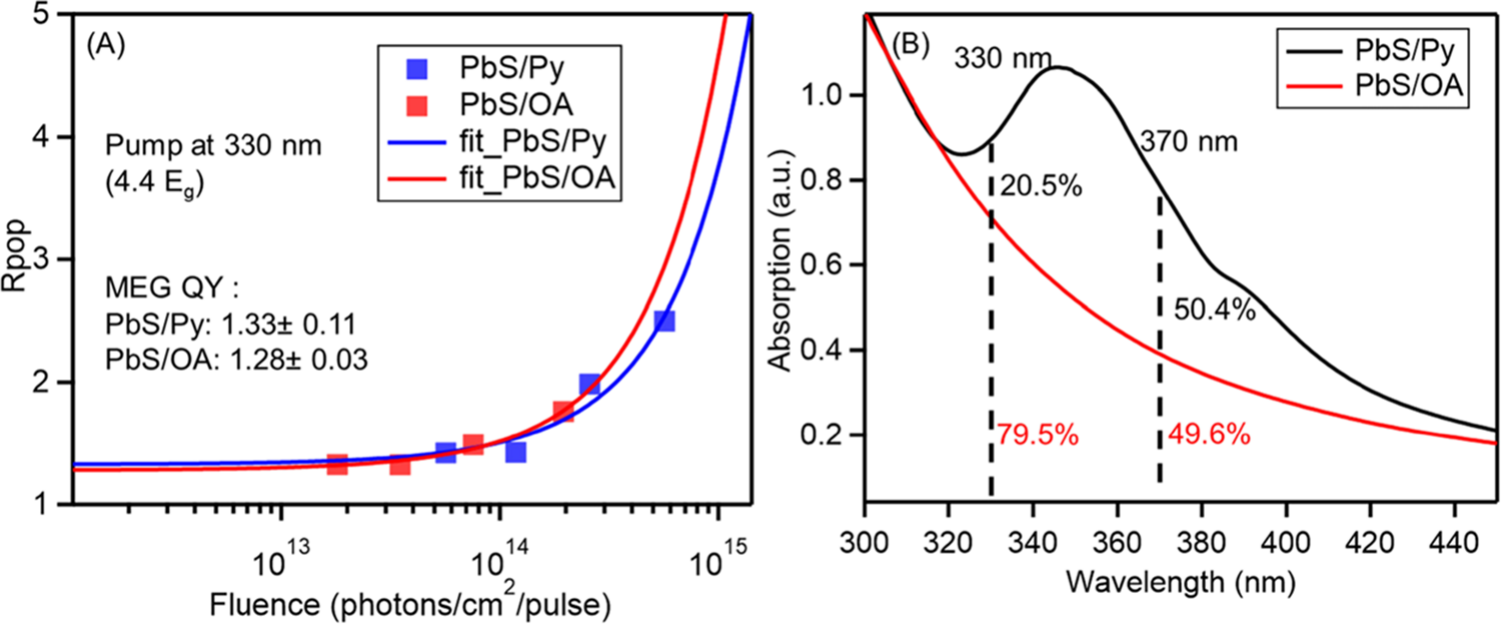
(A) Plot of *R*_pop_ versus the pump photon fluence at 330 nm for PbS/Py (blue) and PbS/OA (red). The solid curves are the fittings with eq 1 to extract MEG QYs. (B) Absorption spectra of PbS/Py (black) and PbS/OA (red) after normalizing at the first excitonic peak. The dashed lines denote the pump positions, and the percentages denote the absorption contributions from pyrene (black) and PbS QDs (red).

We investigated the wavelength dependence of the pyrene-enhanced MEG by using 330 nm excitation light. The results in Figure 3A showed that the MEG QY is 133 ± 11% for PbS/Py and 128 ± 3% for PbS/OA. Thus, only a small enhancement is observed compared with the results with 370 nm pump (Figure 2). This is because the absorption contribution from the pyrene ligands is only 20.5% at 330 nm, but 50.4% at 370 nm, as shown in Figure 3B. Therefore, the QY for the PbS/Py QDs photoexcited with 330 nm is lower than that when photoexciting at 370 nm pump, despite the higher pump-photon energy. In contrast, for the PbS/OA sample, the QY increased from 113 ± 3% to 128 ± 3% when increasing the pump-photon energy from 370 nm (3.9 × *E*_g_) to 330 nm (4.4 × *E*_g_).

To quantify the amount of MEG sensitization, we need to account for the fraction of light that is directly absorbed into core PbS QDs, *f*_QD_, compared to the surface-attached pyrene ligands, *f*_py_. We consider that the measured QY is a linear combination of those QDs that absorbed light directly into the core QD and light absorbed by the pyrene ligands, thus QY = *f*_QD_QY_QD_ + *f*_py_QY_py_. Assuming that QY_QD_ is the same as that measured for the PbS/OA sample, that is, 113% we find that for the 370 nm excitation, where *f*_py_ = *f*_QD_ = 0.5, the QY due to sensitization is QY_py_=183%. For the 330 nm excitation, *f*_py_ = 0.2, and *f*_QD_ = 0.8, we find QY_py_ falls to 153 ± 11%. The lower QY_py_ likely indicates that loss occurs during energy relaxation within the ligands prior to sensitization of the QDs.

**Figure 4:**
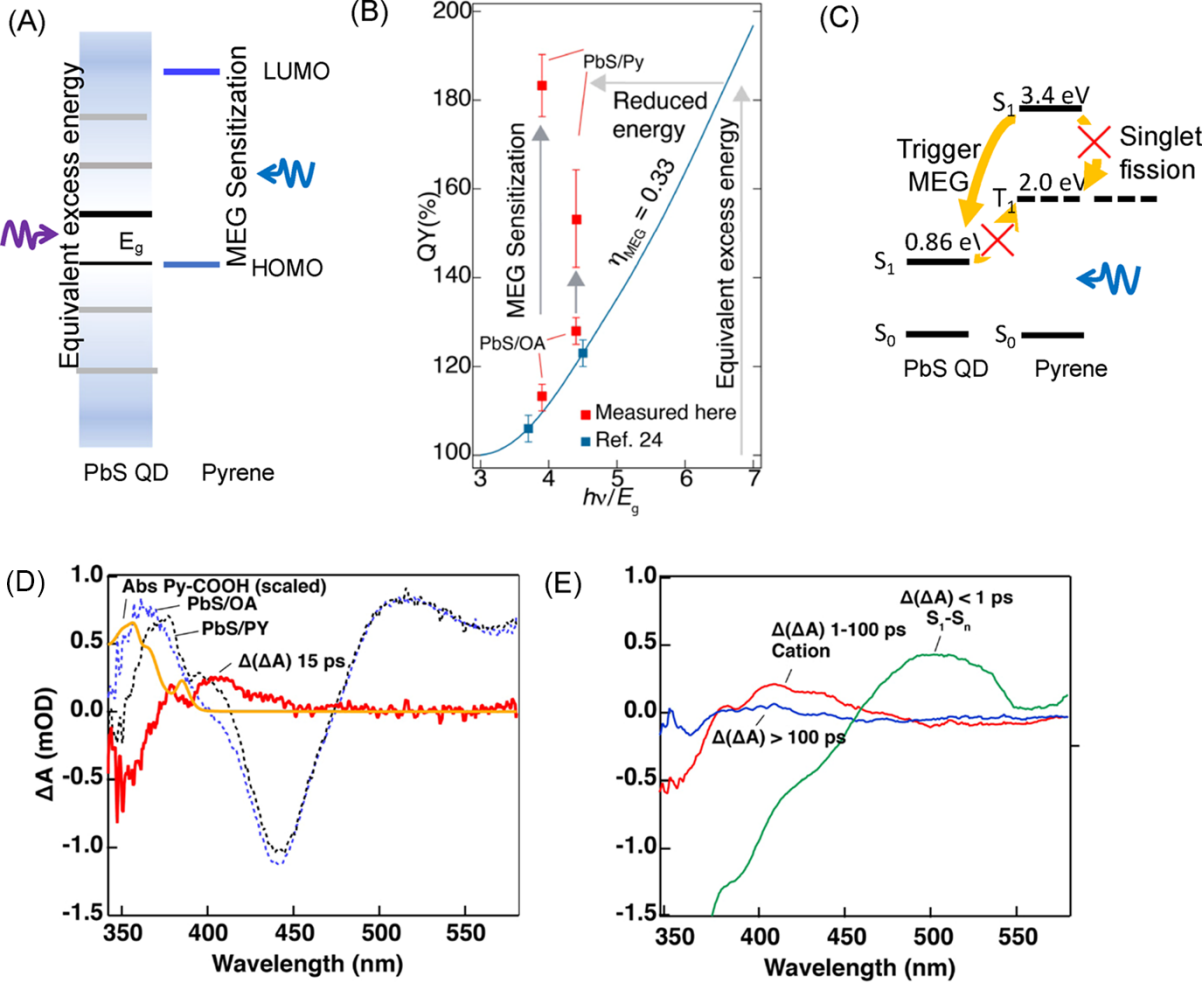
(A) Depiction of MEG sensitization, equivalent excess energy produced either by absorption of high photo energy in the QDs or through absorption within the attached molecule followed by subsequent electron transfer. (B) Comparison of MEG energy efficiency with and without sensitization. (C) Pathway of MEG is initiated through electron transfer and SF is not operable. (D) TA spectra of PbS/OA (blue-trace) and PbS/Py (black-trace), the difference spectra (red-trace) by subtracting the spectrum of PbS/OA from PbS/Py, and the scaled linear absorption spectrum of 1-pyrenecarboxylic acid (yellow-trace). (E) Difference spectra in the three different time regimes: green-trace <1 ps; red-trace 1–100 ps; blue-trace >100 ps.

There are several parameters that are generally reported to characterize MEG: the MEG efficiency (η)^24^ and the electron–hole pair creation energy (ε_eh_, the excess energy to produce an additional exciton).^25^ These parameters can be correlated through:^24,26^
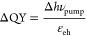
2
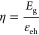
3where the Δ*h*ν_pump_ in eq 2 is the change in pump-photon energy that created the change in QY. With the QYs at 330 and 370 nm, the MEG efficiency η of PbS/OA can be calculated as 0.319, consistent with the reported value of 0.33^24^ for PbS QDs in a similar size range. Considering that the QY of PbS/Py is 1.65 times that for PbS/OA at 370 nm, the effective η and ε_eh_ for PbS/Py are calculated as 0.53 and 1.62, respectively. To our knowledge, 0.53 is the highest reported efficiency for PbS QDs.^4,24^ Assuming the band alignment shown in Figure 4A,C, the excess energy from sensitization corresponds to 2.9 × *E*_g_. Achieving the same amount of excess energy, but directly photoexciting PbS QDs, would require a photon energy of 6.8 × *E*_g_ (Figure 4B) and assuming an η_MEG_ = 0.33 would produce a QY_QD_ of ∼180%, consistent with the enhancement from the sensitization. Thus, sensitization of the MEG process breaks the symmetric excitation within the PbS QDs and increases the energy efficiency of the MEG process.

The underlying mechanism of the pyrene-sensitized MEG effect is unraveled by TA spectroscopy. Figure 4D displays the TA spectra of PbS/OA (dashed blue-trace) and PbS/Py (dashed black-trace) pumped at 330 nm in the 10–20 ps range. The two spectra are well overlapped from 450 to 550 nm and then deviate from 350 to 450 nm. The normalization at 500 nm and then subtraction of the PbS/OA spectrum from the PbS/Py spectrum provide a difference spectrum that represents the contribution associated with the pyrene ligands. In this difference spectrum (Δ(Δ*A*), red-trace), there is a photoinduced absorption (PIA) in the 400–450 nm regime accompanied by a bleach of the pyrene ligands in the 350–400 nm regime that matches the linear absorption of 1-pyrenecarboxylic acid (yellow-trace, scaled). The reported spectral feature of pyrene radical cation in the gas-phase is a narrow peak at 443 nm;^27^ here we assign the PIA in the 400–450 nm range in Figure 4D to the bound pyrene cations whose spectral features are broadened relative to that of the gas phase spectrum. In Figure 4E, we show representative Δ(Δ*A*) spectra in three different time regimes; <1 ps (green-trace); 1–100 ps (red-trace); >100 ps (blue trace). The PIA spectra for the <1 ps in the wavelength range of 480–550 nm are consistent with a *S*_1_–*S_n_* transition and thus represent the initially photoexcited pyrene molecules. This spectral feature rapidly decays within 1 ps, and we assign this process to an ultrafast electron transfer to the QDs leaving behind a pyrene cation (red-trace). After 100 ps (blue-trace), the pyrene molecules have returned to their ground state and have transferred their energy to the QDs (i.e., there is a subsequent hole-transfer). The initial electron transfer initiates the MEG process in the PbS QDs. This conclusion is also supported by the steady-state photoluminescence (PL) spectra. In Figure S4D, the PL intensity of PbS/Py excited at 370 nm is comparable with that excited at 800 nm. The comparable PL intensity for 370 nm excitation and 800 nm excitation indicates that the excited pyrene molecules transfer the energy to PbS QDs with near-unity efficiency. As discussed above, the energy transfer is consistent with a Dexter-like mechanism similar to that observed in other ligand/QD systems.^28−30^ Förster-type energy transfer is less likely in this case because of the weak coupling between QDs and pyrene (Figure 1B). More detailed studies with rationally designed dyes are needed to further unravel the underlying mechanism.

Singlet fission,^31,32^ the analogue of MEG in organic dyes, should be considered when explaining the enhancement of MEG by pyrene. Singlet fission is the generation of a pair of triplets from a singlet excited state and a neighboring ground state, which has been widely observed in acene molecules. The generated triplet excitons are then injected into adjacent QDs so that two excitons are generated from one absorbed photon, the same results as MEG.^33−36^ In this work, each QD has 150 pyrene ligands on the surface (see Figure S5 in the SI for the calculation). While the high surface density meets the requirement for singlet fission, the singlet excited state (*S*_1_) of pyrene is 3.4 eV, lower than twice the triplet state (*T*_1_) 2 × 2.0 eV;^37^ therefore, there is no efficient singlet fission pathway (Figure 4C). Additionally, the thermodynamically unfavorable triplet exciton transportation from PbS QDs to pyrene is hindered. Thus, no triplet excitons should be involved in the pyrene-sensitized MEG process. Consistently, we did not capture any long-lived or short-lived species that correspond to pyrene triplets in the TA spectra.

## Conclusions

In conclusion, we demonstrate the MEG effect of PbS QDs sensitized by surface-anchored pyrene. Using TA spectroscopy, we show that the MEG QY is enhanced from 113 ± 3% to 183 ± 7% when pumping at 3.9 × *E*_g_, which is explained as the result of the rapid-electron transfer from pyrene to PbS QDs producing a highly excited electron in the CB of the PbS QDs. The MEG QY is enhanced in the regime where pyrene has strong absorptivity. Therefore, proper dyes should have strong absorption coefficients at targeted wavelengths and wide absorption bands. Dyes with different band gaps may be used for broad band sensitizations. Additionally, good dye candidates should have appropriate HOMO or LUMO levels with enough excess energy and being asymmetric to the CB and VB of QDs. This work provides a guide to the design of organic dyes to enhance the MEG effect in hybrid nanostructures and promotes the application of MEG in next-generation optoelectronics.

## Experimental Section

### Materials and Instrumentations

Lead nitrate (99.999%), oleic acid (tech. grade, 90%), 1-octene (98%), 1-pyrenecarboxylic acid (97%), and anhydrous solvents of tetrachloroethylene (99%), toluene (99.8%), and acetonitrile (99.8%) were purchased from Sigma-Aldrich. Steady-state absorption spectra were collected using a Cary 7000 spectrometer. Steady-state PL spectra were collected on a Princeton Instruments SpectraPro System with an EnergeTiq laser-driven light source and a PyLoNIR detector. TEM images were obtained on an FEI ST30 transmission electron microscope at 300 kV. TEM samples were prepared by drop-casting diluted solutions on carbon-coated copper grids and dried in air. NMR spectra were recorded on a Bruker Avance 400 MHz spectrometer in deuterated chloroform. The residual solvent peak was used as an internal standard. TA spectra were collected using a PHAROS Yb:KGW laser, with an output of 1028 nm at 1 kHz. The laser beam was directed into an ORPHEUS optical parametric amplifier to generate pump pulse (<190 fs) and was modulated at 500 Hz through an optical chopper to block every other laser pulse. Femtosecond TA spectra were collected using a Helios spectrometer (Ultrafast Systems). A small amount of 1028 nm light was used to pump a CaF_2_ crystal to create 350–600 nm probe light for UV TA or a 1 cm-thick sapphire crystal to generate 1100–1500 nm probe light for NIR TA. Samples were sealed in a 2 mm quartz cuvette in N_2_ glovebox with magnetic stirring during the measurement.

### Sample Preparation

PbS QDs were synthesized by following the reported method,^20^ and the size was determined based on the first excitonic absorption peak.^38^ The ligand exchange reaction was performed in a N_2_ glovebox. In particular, 2 mg of 1-pyrenecarboxylic acid powder was added to 2 mL of toluene containing 1.87 μM PbS QDs. The solution was stirred at 45 °C for 1 h. The resulting solution was filtered with a 0.2 μm PTFE syringe filter and washed three times by precipitation with acetonitrile and redispersion with toluene. After washing, PbS/Py was redispersed in tetrachloroethylene for optical measurements. PbS/OA was prepared following the same ligand exchange and washing procedures mentioned above without adding 1-pyrenecarboxylic acid.

## Supporting Information Available

The experimental details about the NMR spectrum, TEM images, the bi-exciton lifetime, the calculation of the number of pyrene ligands per QD, and the calibration of steady-state PL for the inner-filter effect (PDF)
