# Supplementary material for: Dye-Sensitized Multiple Exciton Generation in Lead Sulfide Quantum Dots
Source: J Am Chem Soc. 2022 Aug 18;144(34):15855–61. doi: 10.1021/jacs.2c07109 (PMC9437916; doi:10.1021/jacs.2c07109)
Supplement: Supplementary file 1 — ja2c07109_si_001.pdf [file ja2c07109_si_001.pdf]

Supporting Information

Dye-Sensitized Multiple Exciton Generation in Lead Sulfide Quantum Dots

Zhiyuan Huang* and Matthew C. Beard*

Chemistry & Nanoscience Center, National Renewable Energy Laboratory, Golden, Colorado, 80401, United States

1. NMR spectra

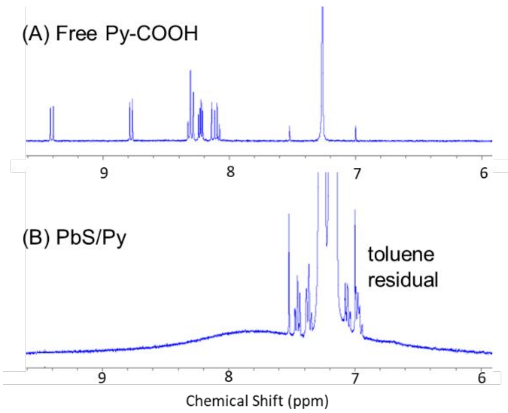

Figure S1. The proton NMR spectra of (A) free 1-pyrenecarboxylic acid, and (B) bound pyrene ligands on PbS QDs in $CDCl_3$. The toluene residual is from the sample preparation.

2. TEM images

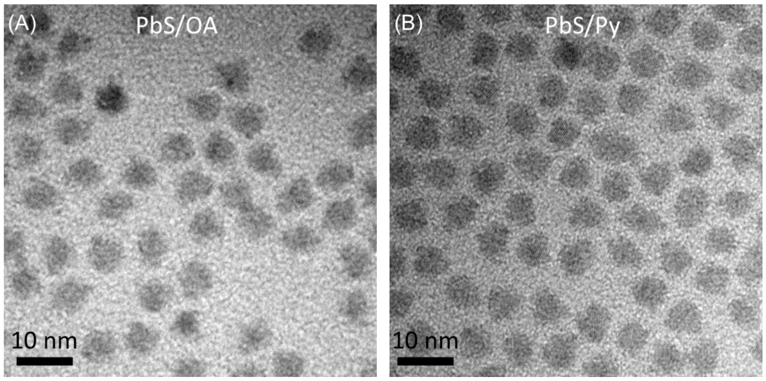

Figure S2. Transmission electron microscope images of (A) PbS/OA and (B) PbS/Py.

3. Extracting biexciton lifetime of PbS QDs

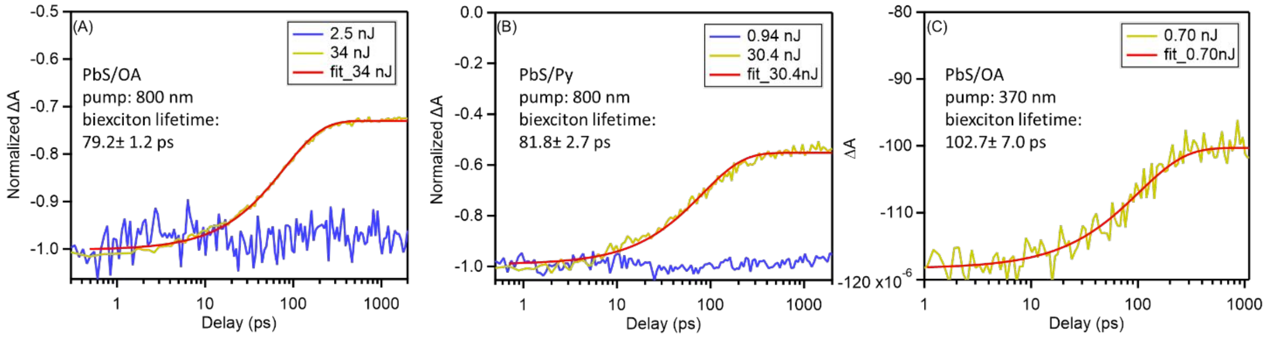

Figure S3. Kinetic traces of the first exciton bleach of (A) PbS/OA, (B) PbS/Py pumped at 800 nm, and (C) PbS/OA pumped at 370 nm. In (A) and (B), the blue curve is obtained at a low pulse energy and the yellow curve is obtained at a high pulse energy. In (C) the yellow curve is obtained

at 0.7 nJ. The red curve is the mono-exponential fitting to the yellow curve to extract the biexciton lifetime.

4. Inner-filter effect calibration

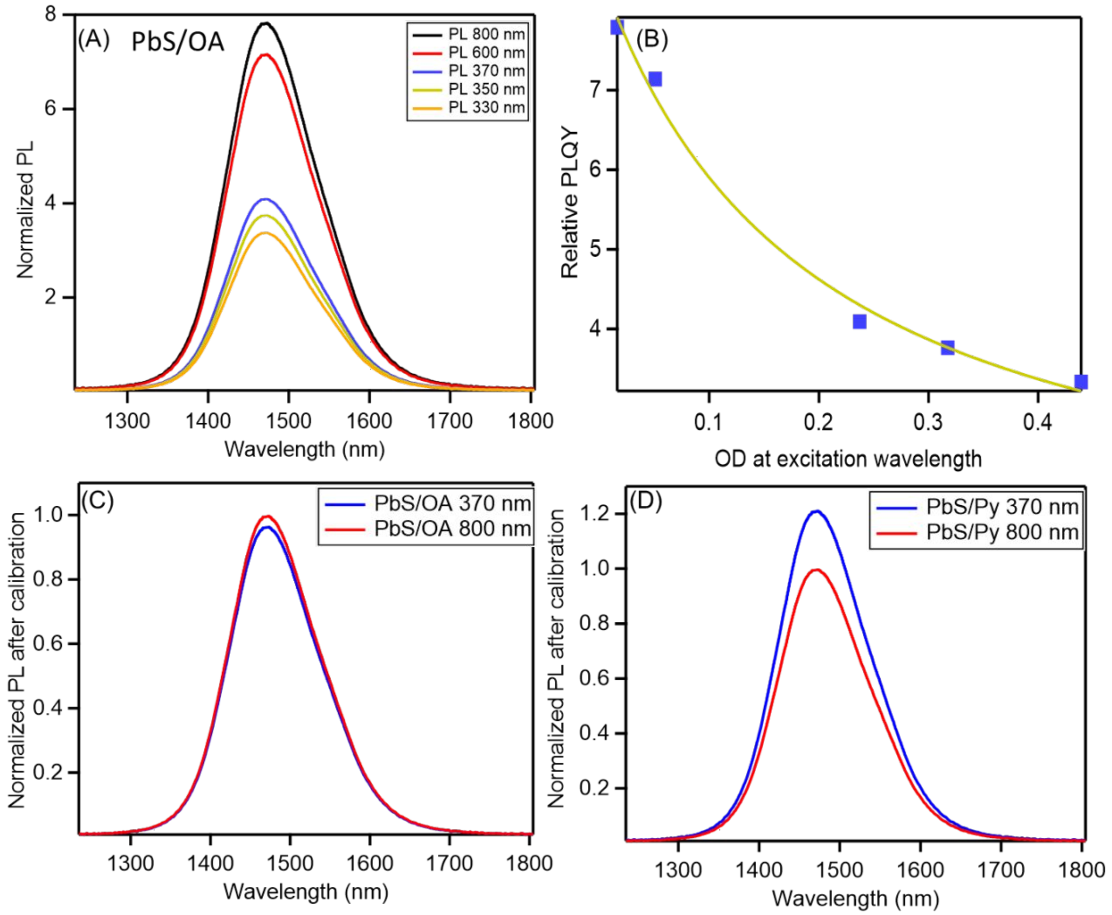

Figure S4. (A) Photoluminescence spectra of PbS/OA with different excitation wavelengths. The spectra are normalized by the ODs and number of incident photons at excitation wavelengths. (B) The observed relative photoluminescence QYs taken from the peak intensity in (A), versus the ODs at excitation wavelengths. The solid curve is the fit with Eq. S4. (C) and (D) are the relative photoluminescence QYs excited at 370 nm (blue) and 800 nm (red) after calibration for PbS/OA and PbS/Py, respectively.

Figure S4 shows the observed relative PL QYs with different excitations. Because the PL is measured with 1cm x 1cm cuvettes, the inner-filter effects lead to lower PL QY when ODs are high. The inner-filter effect can be calibrated with:[1]

$$F_{corr} = F_{obs} * 10^{\frac{A_{ex}+A_{em}}{2}} \quad \text{Eq. S1}$$

therefore,

$$\frac{F_{corr}}{A_{ex}} = \frac{F_{obs}}{A_{ex}} * 10^{\frac{A_{ex}+A_{em}}{2}} \quad \text{Eq. S2}$$

and,

$$PLQY_{corr} = PLQY_{obs} * 10^{\frac{A_{ex}+A_{em}}{2}} \quad \text{Eq. S3}$$

so,

$$PLQY_{obs} = \frac{PLQY_{corr}}{10^{\frac{A_{ex}+A_{em}}{2}}} \quad \text{Eq. S4}$$

where F_{corr} is the corrected PL; F_{obs} is the observed PL; A_{ex} is the OD at excitation wavelength, the x-axis in Figure S4B; A_{em} is the OD at emission wavelength (PL peak maxima); $PLQY_{corr}$ is the corrected PLQY which is a constant; $PLQY_{obs}$ is the observed PLQY, the y-axis in Figure S4B. The calibration curve in Figure S4B is used to calibrate the PL intensity of PbS/Py in Figure S4C-D.

5. Calculate the number of pyrene ligands per PbS QDs

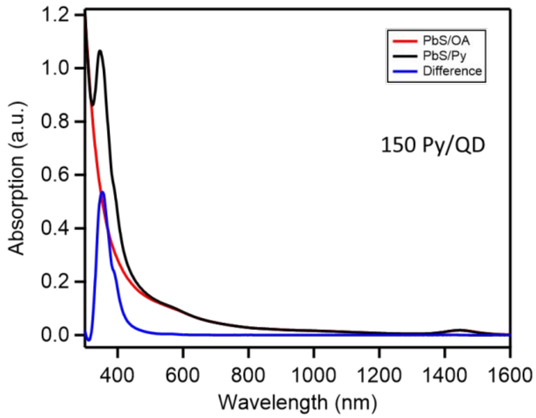

Figure S5. The absorption spectra of PbS/OA (red) and PbS/Py (black) with the normalization at the first excitonic peak, and the difference spectrum by subtracting the spectrum of PbS/OA from PbS/Py. The number of pyrene ligands per PbS QDs in PbS/Py is calculated based on the absorption contribution from PbS QDs (red) and pyrene (blue), as well as the absorption coefficients of PbS QDs[2] and pyrene (measured as 43119 $cm^{-1}M^{-1}$ at 356 nm at the peak maxima).

6. References

(1) Lakowicz, J. R., *Principles of fluorescence spectroscopy*. Springer: 2006.

(2) Moreels, I.; Lambert, K.; Smeets, D.; De Muynck, D.; Nollet, T.; Martins, J. C.; Vanhaecke, F.; Vantomme, A.; Delerue, C.; Allan, G., Size-dependent optical properties of colloidal PbS quantum dots. *ACS nano* **2009**, *3*, 3023-3030.
